# The germination response of *Zea mays* L. to osmotic potentials across optimal temperatures via halo-thermal time model

**DOI:** 10.1038/s41598-024-53129-6

**Published:** 2024-02-08

**Authors:** Fazal Amin, Fakhra Shah, Sami Ullah, Wadood Shah, Iftikhar Ahmed, Baber Ali, Amir Abdullah Khan, Tabarak Malik, Abd El-Zaher M. A. Mustafa

**Affiliations:** 1 Department of Botany, University of Peshawar, Peshawar, 25120 Pakistan; 2 Biological Sciences Research Division, Pakistan Forest Institute, Peshawar, 25120 Pakistan; 3 National Agricultural Research Center, Islamabad, 45500 Pakistan; 4 Department of Plant Sciences, Quaid-i-Azam University, Islamabad, 45320 Pakistan; 5 Institute of Environment and Ecology, Academy of Environmental Health and Ecological Security, Jiangsu University, Zhenjiang, 212013 China; 6 Department of Biomedical Sciences, Institute of Health, Jimma University, 378 Jimma, Ethiopia; 7 Department of Botany and Microbiology, College of Science, King Saud University, 11451 Riyadh, Saudi Arabia

**Keywords:** Plant sciences, Climate sciences

## Abstract

The maize (*Zea mays* L.) is a monocot that is a member of the Poaceae family and a valuable feed for livestock, human food, and raw material for various industries. The halothermal time model determines how plants respond to salt (NaCl) stress under sub-optimal conditions. This model examines the relation between NaClb (g), GR, GP, salinity and temperature stress on germination of seeds dynamics in various crops. Five constant temperatures i.e. 20, 25, 30, 35, and 40 °C and five ψ levels (NaCl concentrations converted to ψ − 0, − 0.2, − 0.4, − 0.6, and − 0.8 MPa) were used in this experiment. In light of the results, the maximum halo-thermal time constant value was recorded at 35 °C temperature, while maximum germination percentage was detected at 30 °C in the controlled condition. Moreover, the lowermost value was recorded at 20 °C at − 0.8 MPa osmotic potential. The highest CAT, APX, and GPX activities were recorded at 15 °C at − 0.8 MPa, while the lowest values were observed for 0 MPa at 30 °C temperature. In conclusion, by employing the halo thermal time model, the germination of maize variety (var.30W52) was accurately predicted for the first time under varying levels of temperature and osmotic potentials.

## Introduction

*Zea mays* L., is a universal cereal plant adapted to diverse agro-ecologies in tropical and temperate region of the world from 40° S to 50° N and from sea level to 4 km height^1^. It is a valuable feed for livestock, human food and raw material for various industries. Maize is native to America but is also grown in other countries including India, Thailand, Pakistan and China as well as various parts of the Philippines. After rice and wheat, maize is the most significant cereal in the world in terms of acreage and total production^2^.

Plant germination is an advanced physiological process in the life cycle of spermatophytes and determines their vital life activities^3^. Naturally, the foremost necessary phases in the growth of a plant are germination and development of seed, which are plague by environmental factors and genetics. In several environments, water accessibility determines seed germination and plant establishment success or failure^4^. The presence of water is necessary for germination, degradation, enzyme activation, transfer, and use of reserve storage materials. Germination percentages and rates increase with water accessibility and reduce with negative water potential. Temperature is a crucial issue poignant germination of seed^5^. Based on specie and environmental condition, wherever they grow, they have three cardinal temperatures [i.e. T_b_-base temperature, which prevents germination, T_o_-optimum temperature, which is when germination occurs most rapidly, and T_c_-ceiling temperature, which does not allow germination]^6–8^.

Germination physiology is complex due to numerous factors, such as optimal environmental conditions, germination rate, and uniformity^9,10^. Biological traits cannot be predicted for a single seed; nevertheless, they are well-established between seed in a population primarily on the basis of population-based threshold models, which include hydro time (HT), thermal time (TT), halotime (HaloT), halothermal time (HaloTT) and hydrothermal time (HTT)^11–13^. As well as quantitatively describing individual diversity on the population degree, these models are also adaptable and may provide explanations for the variety of responses of seed populations^14^.

The halotime model is a powerful tool for measuring the effect of osmotic potential (ψ) specifically soluble salts on seed germination at a specific temperature. This model determines the time required for germination based on the variance between the osmotic potential of the seed environment and the physiological osmotic potential threshold for radicle emergence, also known as the base water potential^11^. Notably, this threshold can differ among seeds in a population, and different germination percentiles correspond to different base water potential values. Base water potential values in seed populations typically follow a normal distribution. Ultimately, the halotime model is invaluable for analyzing the germination process, identifying factors that impact it, and ultimately enhancing crop yields and optimizing plant growth^15^.

The seed germination model known as hydrothermal time (HTT) is a broadly acknowledged theory elucidating the germination process influenced by temperature and water potential. According to this model, germination occurs when the seed's temperature (Ts) falls within the range of the base temperature (Tb) and the optimal temperature (To), while maintaining a constant water potential (ψ). The model posits that the germination time (tg) for specific percentiles of germination in a seed population is influenced by the extent to which temperature and water potential surpass their respective base values, Tb and ψb^16^.

At first, the HTT model presumed a consistent and uniform thermal time requirement (θHT) and Tb for all seeds, with only variations in ψb(g) within the seed population. However, this initial model overlooked the inhibition of seed germination, encompassing both germination rate (GR) and germination percentage (GP), when Ts surpassed To. In response, Alvarado and Bradford^17^ proposed an alternative model that accounted for this limitation. In their model, they propose that the correlation between GR and temperature exhibits a negatively linear trend when Ts surpasses To, where a universal optimal temperature (To) exists for all germination percentiles, but critical temperature (Tc) values differ within the seed population. This model offers a more precise and comprehensive explanation of seed germination under varying temperature and water potential conditions. The linear elevation in ψb(g) as temperature exceeds To is attributed to the observed decline in both GR and GP. Alvarado and Bradford (2002) introduced a constant parameter (kT) to represent the incremental change in ψb(50) per degree when the temperature surpasses To. This modeling approach has effectively foretold seed germination in various crops such as sesame^18^, watermelon^19^, potato^17^, and arugula^20^.

Hydrothermal time models are threshold models that concurrently account for germination percentages and seeds' germination rates^21^. In those boundaries, seeds generally germinate faster in moister, hotter environments. In this concept, seeds germinate when they accumulate enough moisture and heat^6^. On the basis of the hydrothermal time model, Seal, et al.^22^ developed the halothermal time model to determine how *Suaeda maritima* responds to salt (NaCl) stress under sub-optimal conditions. This model examines the relation between NaClb (g), GR, and GP, Salt and Temperature stress on germination of seeds dynamics in various crops are simultaneously quantified using this HaloTT model as a vital framework^23^. Evaluation of the functional factors of germination is challenging as several factors are required such as: rate, consistency, and percentage of germination under optimal and stress-inducing environmental situations^24^. It is the first time study of maize in Pakistan via halothermal time model.

The goals of this study were: (1) to examine the effect of temperature and salt stresses on maize seed germination and ROS scavenging enzymes; (2) to evaluate the cardinal temperatures for germination using the HaloTT model; and (3) to quantify the amount of osmotic adjustment due to sodium chloride absorption.

## Materials and methods

### Seed germination and experimental protocol

Seeds of *Zea mays* L. variety (var.30W52) was obtained from NIFA (Nuclear institute of food and agriculture), Pakistan, with a viability rate of 90%. The treatments included five constant Ts (20, 25, 30, 35 and 40 °C), and five ψ levels (NaCl concentrations converted to ψ were 0, − 0.2, − 0.4, − 0.6 and − 0.8 MPa). To verify the osmotic potential for every temperature, an osmometer (model 5520: Wescor Inc., USA) was used. Salt stress levels were prepared using sodium chloride (NaCl). Using the Van’t Hoff equation^25^, NaCl(M) concentrations at each temperature T were changed into water potential (Ψ) and then confirmed on an osmometer as well. Each triplicate treatment consisted of thirty seeds per petri dish on two layers of Whitman No.1 filters papers in a 10 cm petri-dish with 6 ml of each solution. Petri-dishes were arranged at random in dark besides the duration of recording in the incubator with a temperature accuracy of ± 0.5 °C. The seeds were observed at regular intervals and were deemed germinated when the radicles reached 2 mm in length. Afterward, seeds were extracted, and germination parameters were assessed.

### Data analysis

#### Halothermal time model

The maize seeds which have been germinated with different variety of Temperatures (T) and water potential (ψ) accompanied with the aid of using a well-known model referred to as Halothermal time (HaloTT) Model. In order to determine the parameters for the Halo-time, thermal time models and Halothermal time repeated Probit regression analysis was used^15,18,26^.

#### Thermal time model (TT)

Based at the HaloTT model idea, Sub and Supra optimal Ts have been derived from the beneath formulas^27^.

1 $${\text{TTsub}}=\left({\text{T}}-{\text{Tb}}\right)\,\,\,\,\mathrm{tg \,\,\,\,at\,\,\, sub}-\mathrm{optimal \,\,\,T},$$

2 $${\text{TTsupra}}={\text{Tc}}({\text{g}})-{\text{T}})\,\,\,\,\mathrm{tg\,\,\, at \,\,\,supra}-\mathrm{optimal \,\,\,T}.$$

#### Halotime model (θHalo)

It determines solute potential (NaCl) and rate of germination (tg) similar to thermal time model^20^:

3 $$\mathrm{\theta Halo}=(\mathrm{NaClb }-\mathrm{ NaCl})\mathrm{ tg}.$$

#### Halo-thermal time model (HaloTT)

The halo-thermal time model can estimate tg at every water potential and Temperature (T) in the sub-optimal Ts^20^.

4 $$\mathrm{\theta HaloTT }= \left({\text{NaClb}}-{\text{NaCl}}\right)\left(\mathrm{T }-{\text{Tb}}\right){\text{tg}},$$

5 $${\text{Probit}}({\text{g}}) = [({\text{NaCl}}(\frac{\mathrm{\theta HaloTT}}{{\text{T}}-{\text{Tb}}}{\text{tg}})-{\text{NaClb}}(50)]/\mathrm{\sigma NaClb}.$$

### Germination and agronomic parameters

The below-cited morphological indices had been calculated from the length (radicle, plumule and leaf), germination rate, fresh weight (radicle, plumule and leaf), and dry weight (radicle, plumule and leaf).

#### Germination percentage (G%)

The G% illustrates the number of seed that emerged from all the seeds implanted in each pot. In this case, the germination factor was determined using Irshad, et al.^28^ formula.

6 $$Germination \,\,percentage=\frac{\mathrm{Final \,\,number \,\,of \,\,seedlings \,\,emerged}}{\mathrm{Total \,\,number \,\,of \,\,seeds }}\times 100.$$

#### Germination energy (GE)

To calculate germination energy, Maguire^29^ formula was used.

7 $$\mathrm{GE }=\frac{X1}{Y1}+\left(\frac{X2-X1}{Y2}\right)+\left(\frac{Xn-Xn-1}{Yn}\right).$$

#### Germination index (GI)

Germination indexes provide details on speed of germination and germination percentage. To calculate the GI, we used a methodology developed by Hafez et al.^30^.

8 $${\text{GI}}=\left(10 *{\text{n}}1 \right)+\left(9\mathrm{x n}2\right)\dots + \left(1\mathrm{ x n}10\right).$$

#### Germination rate index (GRI)

GRI measures the percentage of germination at a given time and day. In order to calculate it, use the following formula of Kader^31^.

9 $${\text{GRI}}=\frac{{\text{G}}1}{1}+\frac{{\text{G}}2}{2}+\frac{{\text{G}}3}{3}\dots .\frac{{\text{Gx}}}{{\text{x}}}.$$

#### Timson germination index (TGI)

Timson germination index measures the mean number of seeds emerged each day. We can estimate by applying the mathematical formula given by Ullah et al.^27^.

10 $${\text{TGI}}=\frac{\in {\text{G}}}{{\text{T}}}.$$

In this equation, G is the total percentage of germination on each day, and T is the germinations time.

#### Mean germination time (MGT)

MGT measures how quick seed emerge in a population. When MGT is small, the population of seed has a high rate and vice versa. As determined by the formula of Orchard^32^.

11 $$\mathrm{MGT }= \frac{\in {\text{fx}}}{\in {\text{f}}}.$$

On day X, f represents the No. of seeds that germinated.

#### Mean germination rate (MGR)

The mean Germination Rate is the inverse of the mean germination time, and has been determined by the Ranal and Santana^33^ according to following formula.

12 $${\text{MGR}} =\frac{1}{{\text{MGT}}}.$$

#### Coefficient of velocity of germination (CVG)

The rate of seed germination is represented by CVG in an experiment. Increased germination frequency will increase this number. When every sprouted seed germinate on the 1st day, the CVG value reaches its highest theoretical value. This is calculated using Maguire^29^ formula:

13 $${\text{CVG}}=\frac{N1+{\text{N}}2+{\text{N}}3\dots {\text{Nx}}}{100}\times {\text{N}}1{\text{T}}1\dots .{\text{NxTx}}.$$

#### Time to 50% germination (T50%)

T50% was used to measure the time needed for the 50% of seeds to germinate. This is stated via a mathematical formulation given by Ullah et al.^27^

14 $${\text{T}}50\mathrm{\%}=\frac{{\text{Ti}}+\left(\frac{{\text{N}}}{2}-{\text{ni}}\right)\left({\text{Tj}}-{\text{Ti}}\right)}{\left({\text{nj}}-{\text{ni}}\right)}.$$

#### Seed vigor index − I (SVI − I)

From every pot were three plantlets estimated in centimeter and then measured using the Uddin et al.^34^ formula.

15 $${\text{SVI-I}}={\text{SL}}\left({\text{cm}}\right)\times {\text{SG}}.$$

In which SL stand for seedlings length and SG stand for seed germination.

#### Seed vigor index − II (SVI − II)

The formula of Uddin et al.^34^ was used to determine the SVI-II.

16 $${\text{SVI-II}}=Seed \,\,dry\,\, weight \left(mg\right)\times Seed \,\,Germination.$$

### Anti-oxidant enzymes activities

#### Guaiacol peroxidase peroxidase activity (GPX)

The experimental procedure involved creating a reaction mixture using fresh plant tissue. Initially, 0.5 g of fresh plant tissue underwent homogenization in 10 ml of phosphate buffer. Following homogenization, the mixture underwent centrifugation, and the resulting supernatant was collected. Subsequently, 0.1 ml of the supernatant were combined with 16 mM guaiacol and 50.0 mM phosphate buffer. To this mixture, 2 mM H_2_O_2_ was introduced. The total volume of the reaction mixture was then adjusted to 3.0 ml by incorporating de-ionized water. Ultimately, the absorbance of the resultant mixture was measured at 470 nm, employing the protocol outlined by Hussain et al.^35^.

#### Ascorbate peroxidase assay (APΟX)

The assessment of ascorbate peroxidase followed the methodology outlined by Shah et al.^36^. Initially, a 0.5-g sample of fresh leaf material was ground in 5 ml of phosphate buffer with a pH of 7. Following centrifugation for 15 min, 0.2 ml of the resulting supernatant were collected. The collected supernatant underwent treatment with 0.10 mM hydrogen peroxide, 0.6 mM ascorbic acid, and 0.1 mM ethylene diamine tetraacetic acid (EDTA).

#### Determination of catalase assay (CAT)

Catalase activity assessment followed the protocol outlined by Ullah et al. Ullah et al.^37^. In a 5.0-ml buffer solution with a pH of 7.0, 0.5 g of fresh foliar material were homogenized. After centrifuging the mixture at 3000.0 rpm for 15 min, 0.1 ml of the resulting supernatant were extracted. Subsequently, 1.90 ml of phosphate buffer (50 mM) and 0.10 ml of H_2_O_2_ (5.90 mM) were introduced to the supernatant. The optical density (OD) was recorded at 240 nm.

#### Estimation of superoxide dismutase activity (SOD)

A standard methodology Ullah and Bano^38^ was used to calculate SOD activity. In phosphate buffer, the homogenization and centrifugation of plant material (0.5 g). A 0.1 ml filtrate was then obtained by combining 24 l of nitro blue tetrazolium, riboflavin and methionine. OD (Optical density) at 560 nm was measured after 3 min.

17 $${\text{SOD}}=\frac{{\text{ODcontrol}}-{\text{ODtest}}}{{\text{ODcontrol}}}\times \frac{1}{50}\times \frac{{\text{Vt}}}{{\text{SQ}}}\times {\text{FW}}.$$

#### Quantification of peroxidase (POD) activity

The POD activity was determined using a standard method^39^. The supernatant was collected after centrifuging plant sample (0.50 g) in MES (morpholine ethane sulfonic acids). In the supernatant, phenyl diamine, MES, and 30% H2O2 were added. At 470 nm, the optical density was measured.

18 $${\text{POD}}=\frac{\mathrm{change \,\,in\,\, OD}}{\mathrm{time\,\, taken}}\times \frac{1}{{\text{EC}}}\times \frac{{\text{TV}}}{{\text{UV}}}\times \frac{1}{{\text{FW}}}\times 100.$$

### Statistical analysis

Statistical analysis were conducted using IBM SPSS Statistic 26, Excel, and ORIGIN 2021 from PC corporation. In our study, we employed various statistical analyses to assess the impact of salinity and temperature (T) on seed germination. Specifically, we utilized Analysis of Variance (ANOVA) along with the LSD (least significant difference) test for mean comparisons, correlation analysis, and Principle Component Analysis (PCA). These tests enabled us to thoroughly evaluate the relationships and outcomes of temperature and salinity on the germination process. Heat map was created by using Origin Pro (2021b) (https://www.originlab.com/2021b?go=Products/Origin/2021b&pid=4416).

### Ethics approval and consent to participate

The Seeds of *Zea mays* L. var. 30W52 were acquired from NIFA (Nuclear institute of food and agriculture), Pakistan All the experiments were performed in accordance with relevant guidelines and regulations".

## Results

### Effect of water potentials and temperatures on agronomic parameters

Initially the rise in T (temperature) favoured the germination rate (GR) and germination percentage (GP) but decrease in the value of germination percentage occur when the temperature exceeded a particular limit. The σψb (standard deviation) value was relatively stable on every temperatures. The maximum σψb is recorded at 25 $^\circ{\rm C}$, while the lowest was observed at 20 $^\circ{\rm C}$ (Table 1). Furthermore, the most increased Ψb at 50% germination was found at 35 °C (− 0.97 MPa). Germination is influenced by three temperature levels: a Tb (base temperature) below which germination declines, an optimum temperature (To) at which germination occurs most rapidly, and a ceiling temperature (Tc) that suppresses germination when surpassed. For Tb, To, and Tc, the HTT concept predicted cardinal temperature of 20 °C, 33 °C, and 45 °C, respectively (Table 2). According to the results in Table 3 the GR of maize is highly influenced by temperature. The GR was maximum at 25 $^\circ{\rm C}$ and minimum at 40 $^\circ{\rm C}$. The Germination increase initially when temperature increase upto 30 $^\circ{\rm C}$ (optimum temperature) and decrease with further increase in temperature. In light of the results, a very highest value of TTsub was recorded in 40 °C at 0 MPa and the greatest value of TTsupra was documented in 0 MPa at 20 °C. The GP was designed against various temperature percentile, and the results demonstrated that the germination rate demonstrated a linear increase both above and below the optimum temperature (To). The maximum halo-thermal time constant (θHalo) value was recorded at 35 $^\circ{\rm C}$. Further, GR value show a significant (p < 0.01) improvement with a reduction in Ψ at all temperatures (Table 3).

**Table 1:** Halo thermal time (HaloTT) model-based predictions of R2, ψb(50), and σψb for Zea mays L. var. 30W52 seed germination under varied temperature (Ts) and water potential (ψs) conditions..

Temperature	*ѱ*b(50) (MPa)	σ*ψ*b (MPa)	*R*^*2*^	SE	F	T	Sig
20˚C	− 0.83	0.27	0.407	3.51	127.43	23.85	0.001
25˚C	− 0.76	0.36	0.318	4.19	145.30	22.20	0.001
30˚C	− 0.96	0.29	0.203	3.61	120.62	25.12	0.002
35˚C	− 0.97	0.29	0.348	4.08	72.36	22.12	0.001
40˚C	− 0.82	0.30	0.451	4.43	73.99	20.02	0.003

R and R2 represent the coefficient of determination, while σψb denotes the standard deviation. Ψb(50) corresponds to the base water potential at the 50th percentile, and θH stands for the halotime constant. F indicates the variability between different means, and Sig. indicates the significance value.

**Table 2:** Using the halothermal time model (HaloTT), the values of kT, σψb, and To estimated for the seed germination of Zea mays L. var. 30W52 under varying Ts and ψs.

Variables	Zea mays
Hydrothermal time model parameters	
*Ѱ*b (50) (MPa)	− 0.87
σ*ψ*b (MPa)	0.30
θH (MPa˚Ch-1)	56.29
kT (MPa˚Ch-1)	0.104
Cardinal temperatures	
*T*b ($^\circ{\rm C}$)	20
*T*o ($^\circ{\rm C}$)	33
*T*c ($^\circ{\rm C}$)	45
*R*^*2*^	0.885

*Tb* base temperature, *To* optimum temperature, *Tc* ceiling temperature, Ψb(50) base water potential at 50th percentile.

**Table 3:** The parameters for the halo and thermal time model that describe the germination of Zea mays L. var. 30W52 seeds under varying temperatures (Ts) and water potentials (ψs) have been estimated.

Temperature	Ψ (MPa)	TTsub	TTsupra	θHalo (MPa h)	θHaloTT (MPa h)	TT GR	HT GR
20 $^\circ{\rm C}$	0 Mpa	222.40	1334.40	66.72	333.60	0.022	0.022
	− 0.2 Mpa	216.00	1296.00	65.10	259.20	0.023	0.018
	− 0.4 Mpa	160.00	960.00	47.40	144.00	0.032	0.019
	− 0.6 Mpa	138.40	830.40	40.62	83.04	0.036	0.015
	− 0.8 Mpa	65.60	393.60	19.28	19.68	0.078	0.016
25 $^\circ{\rm C}$	0 Mpa	484.80	1212.00	72.72	727.20	0.021	0.021
	− 0.2 Mpa	470.40	1176.00	70.56	564.48	0.021	0.017
	− 0.4 Mpa	326.40	816.00	48.96	293.76	0.031	0.019
	− 0.6 Mpa	163.20	408.00	24.48	97.92	0.063	0.025
	− 0.8 Mpa	131.20	328.00	19.68	39.36	0.076	0.015
30 $^\circ{\rm C}$	0 Mpa	840.00	1120.00	84.00	1260.00	0.018	0.018
	− 0.2 Mpa	602.40	803.20	60.24	722.88	0.025	0.020
	− 0.4 Mpa	511.20	681.60	51.12	460.08	0.029	0.018
	− 0.6 Mpa	487.20	649.60	48.72	292.32	0.031	0.012
	− 0.8 Mpa	324.00	432.00	32.40	97.20	0.046	0.009
35 $^\circ{\rm C}$	0 Mpa	1104.00	828.00	82.80	1656.00	0.018	0.018
	− 0.2 Mpa	924.80	693.60	69.36	1109.76	0.022	0.018
	− 0.4 Mpa	1881.60	1411.20	141.12	1693.44	0.019	0.012
	− 0.6 Mpa	672.00	504.00	50.40	403.20	0.030	0.012
	− 0.8 Mpa	540.80	405.60	40.56	162.24	0.037	0.007
40 $^\circ{\rm C}$	0 Mpa	1304.67	521.87	78.28	1957.00	0.019	0.019
	− 0.2 Mpa	1064.00	425.60	63.84	1276.80	0.024	0.019
	− 0.4 Mpa	902.00	360.80	54.12	811.80	0.028	0.017
	− 0.6 Mpa	752.00	300.80	45.12	451.20	0.033	0.013
	− 0.8 Mpa	668.67	267.47	40.12	200.60	0.038	0.008

Germination rate (GR), halo-thermal time constant (θHTT), halotime constant (θH), thermal time constant at supra-optimal temperature (TTsupra), thermal time constant at sub-optimal temperature (TTsub), temperatures (T), water potential (ψ).

**Figure 1:**
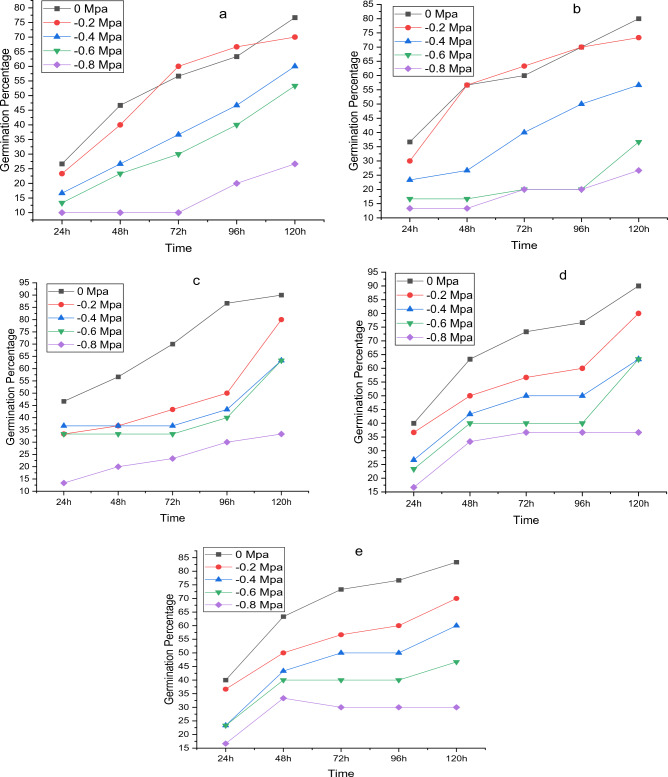
*Zea mays* L. var. 30W52 germination at (**a**) 20 °C, (**b**) 25 °C, (**c**) 30 °C, (**d**) 35 °C and (**e**) 40 °C having various water potentials.

The germination process (GP) is significantly affected by water potential (Ψ) and temperature. Under control, the highest percentage of germination occurred at 30 °C, whereas the lowest percentage was observed at 20 °C with a water potential of − 0.8 MPa. The germination percentage initially increased with increase in temperature but decrease after increasing temperature from 35 $^\circ{\rm C}$. Osmotic potential also negatively affected the germination percentage. Increase (more negative) in osmotic potential (Ψ) decrease the germination percentage (Fig. 1a–e). Recent Halothermal time model experiments showed that NaCl and temperature greatly affected the germination factors of Maize (*Zea mays* L.).

**Figure 2:**
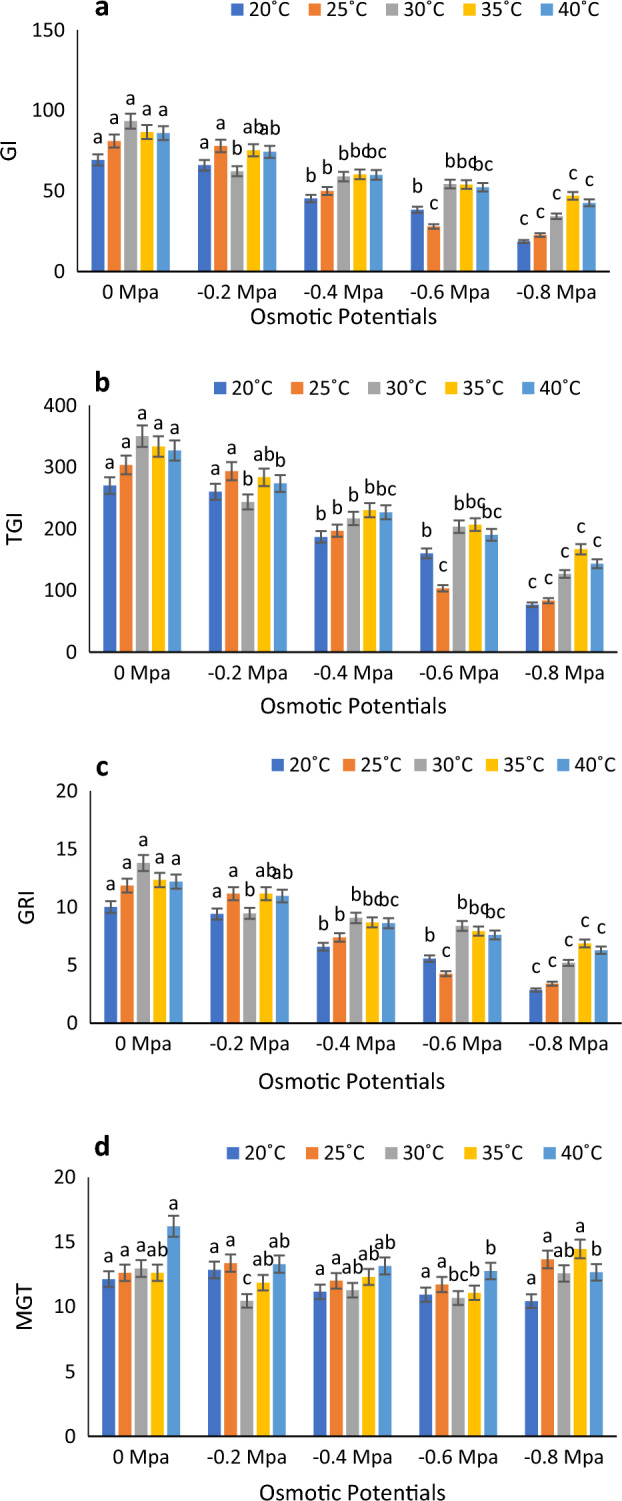
Combined effect of temperature and water potential on (**a**) germination index, (**b**) Timson germination index (**c**) germination rate index and (**d**) mean germination time of *Zea mays* L. var. 30W52 using halothermal time mode.

**Figure 3:**
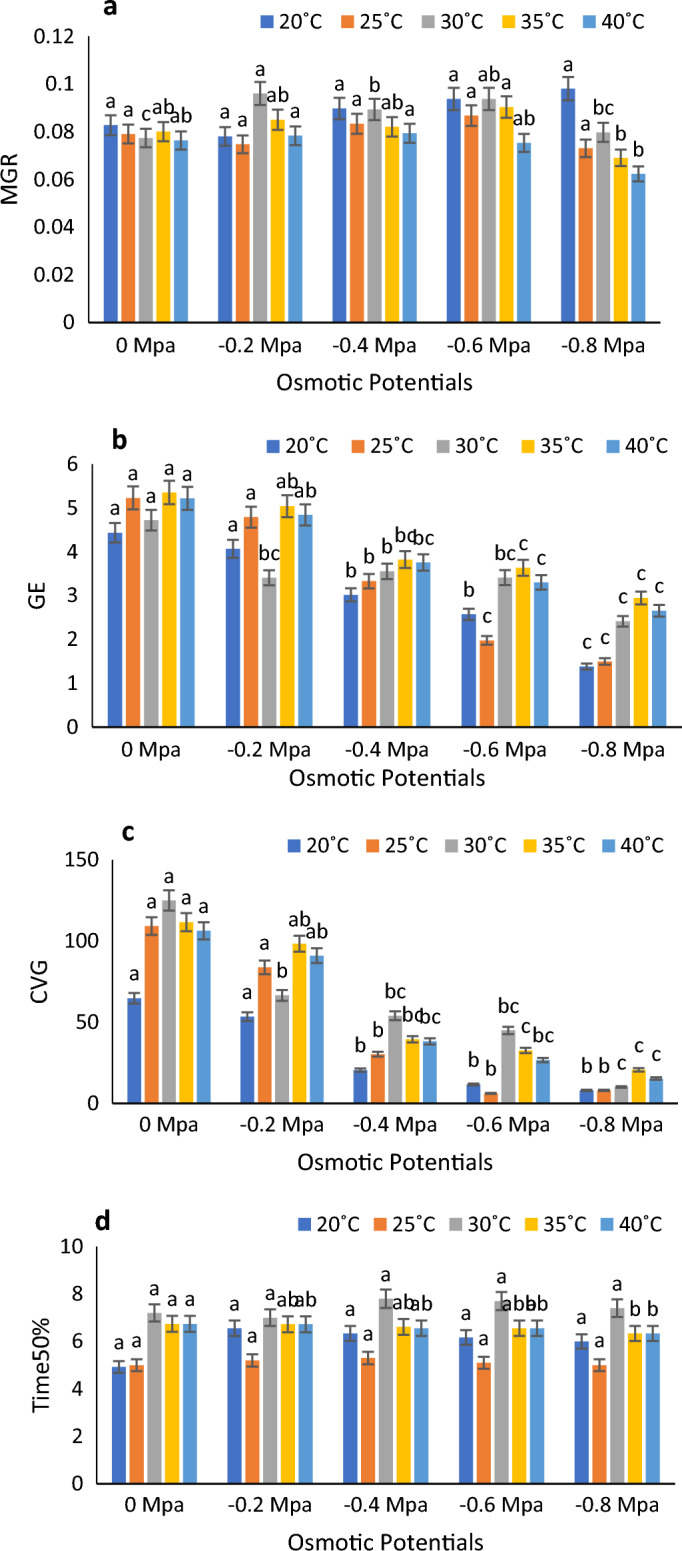
Combined effect of temperature and water potential on (**a**) Mean Germination Rate, (**b**) Germination Energy (**c**) Coefficient of velocity of Germination and (**d**) Time to 50 percent Germination of *Zea mays* L. var. 30W52 using Halothermal time model.

**Figure 4:**
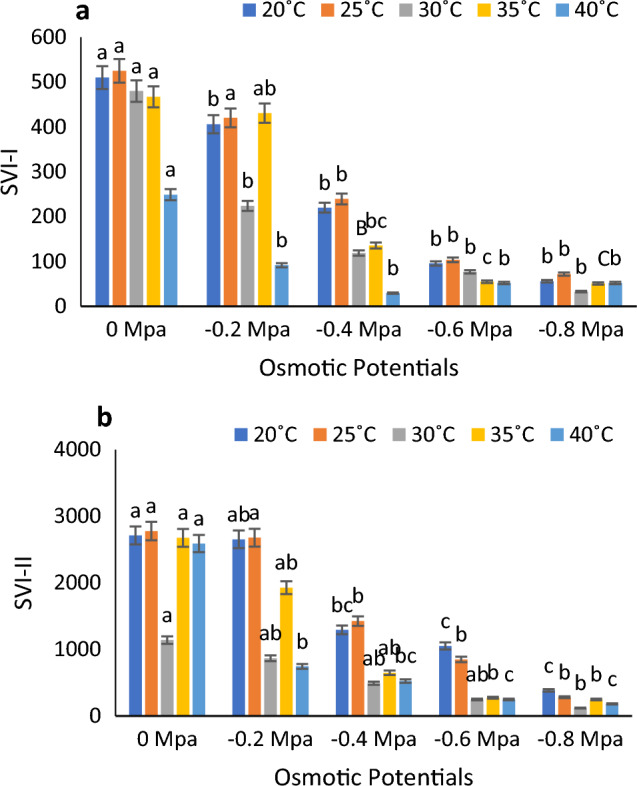
Combined effect of water potential and temperature on (**a**) seed vigor index-I and (**b**) seed vigor index-II of *Zea mays* L. var. 30W52 using Halothermal time model.

At 30 degrees Celsius in distilled water with a water potential of 0 MPa, Timson germination index (TGI), GI (germination index), and GRI (germination rate index) reached their peak values. Conversely, at 20 degrees Celsius with a water potential of 0.8 MPa, they exhibited their lowest values. Meanwhile, mean germination time (MGT) was recorded at its highest at 40 °C with a water potential of 0 MPa, and its minimum value was observed at 20 °C with a water potential of 0.8 MPa. (Fig. 2a–d). According to Fig. 3a, the lowest and highest MGT were registered at 40 °C and 30 °C, respectively. Figure 3b shows the highest and lowest Germination energy (GE) values at 35 $^\circ{\rm C}$ and 20 °C at 0 MPa and − 0.8 MPa. CVG and time to time50% (50% germination) were both highest at 30 °C in control and 0.4 MPa, respectively (Fig. 3c and d). According to Fig. 4a and b, SVI − I and SVI − II were maximum at 25 °C at 0 MPa and minimum at 40 °C at 0.8 MPa.

### Results of antioxidant enzymes under different water potentials and temperatures

**Figure 5:**
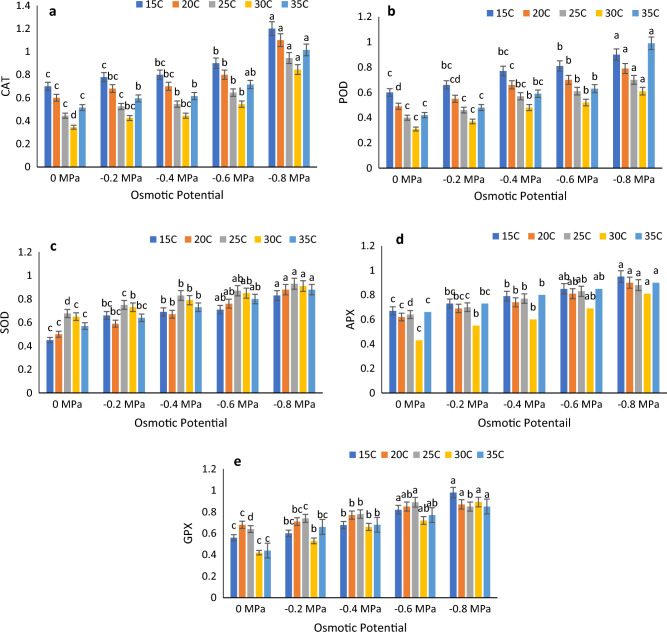
Combined effect of temperature and water potential on (**a**) CAT, (**b**) POD (**c**) SOD (**d**) APX and (**e**) GPX of *Zea mays* L. var. 30W52 using Halothermal time model.

Antioxidant enzymes of fresh plant tissues were significantly affected by fluctuating temperatures and osmotic potentials. The highest CAT, APX and GPX activities were recorded in 15 $^\circ{\rm C}$ at − 0.8 MPa, while the minimum values were recorded for 0 MPa at 30 $^\circ{\rm C}$. Based on the results, the POD enzyme demonstrated its highest activity at − 0.8 MPa while the lowest activity was observed in the control group at 30 $^\circ{\rm C}$. Furthermore, the SOD enzyme exhibited its greatest activity at 30 $^\circ{\rm C}$ and − 0.8 MPa, while the lowest activity was recorded at 15 $^\circ{\rm C}$ and 0 MPa. Interestingly, all enzymes appeared to respond normally in the control group at 0 MPa, with the lowest response observed across all antioxidant enzymes under these conditions. However, it should be noted that exposure to both high and low temperatures had a significant impact on the activity of antioxidant enzymes (Fig. 5a–e).

### PCA and Correlation of germination parameters and antioxidants enzymes of maize to fluctuating water potentials and temperatures

**Figure 6:**
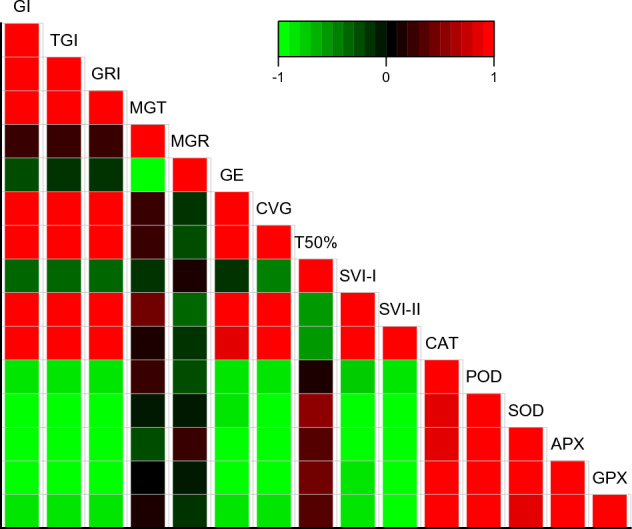
Using the Halothermal time model to determine the correlation between different germination attributes of *Zea mays* L. var. 30W52.

**Figure 7:**
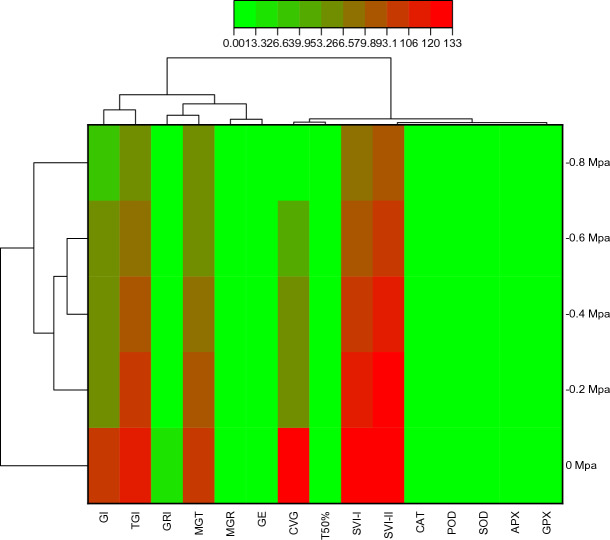
Heatmap histogram correlation between different germination attribute of *Zea mays* L. var. 30W52 using Halothermal time model.

**Figure 8:**
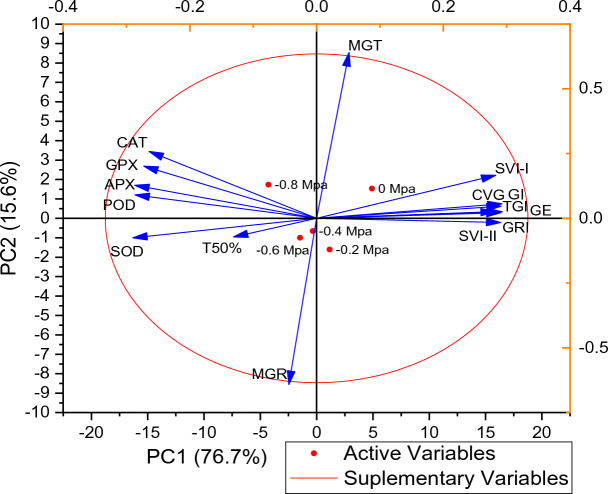
Loading plot of principal component analysis (PCA) on different germination attributes of Zea mays L. var. 30W52 using Halothermal time model.

From correlation analysis it was observed that antioxidant enzymes were positively correlated to each other while negatively correlated with GI, SVI-I, SVI-I, TGI, GE, GRI, and CVG. T50% was negatively correlated with germination parameter and positively correlated with antioxidant enzymes. MGT and MGR showed a negative correlation with germination parameters as well as with antioxidant enzymes (Fig. 6). Based on Heat-map correlation analysis, it was found that two separate clusters were formed among the treatments. To analyze the germination dataset, PCA was utilized. The first cluster comprised of 0 MPa and the second cluster comprised of − 0.2, − 0.4, − 0.6, and − 0.8 MPa (as shown in Fig. 7). It was observed that the treatments were distributed throughout the dataset, indicating that osmotic potentials significantly influencing the rate of germination. Moreover, the PCA results demonstrated that the first two components accounted for 76.7% of the total variance. The highest variation found in the first two components so a PCA bi-plot was constructed (Fig. 8).

## Discussion

Accurately predicting the timing of seed germination is an essential aspect of managing crops, especially when facing challenging conditions such as drought, heat, and salinity^40^. In the case of maize crops, the use of high-quality seed is critical to producing a healthy and productive crop. Therefore, it is crucial to enhance our ability to predict the germination response of maize seed with varying levels of vigour to environmental changes that may occur in the future. In field conditions, the timing of germination can be affected by several factors, including the properties of the seed, soil, and environment^41–43^. Thus, having a reliable model that can accurately predict the timing of seed germination is highly beneficial for farmers and decision-makers in the agricultural industry. By leveraging such a model, farmers can make informed decisions about crop management and optimize their yields, ultimately leading to better food security and economic growth.

HaloTT accurately represents the actual responses of GRs and GPs. Studying seed germination under changing environmental situations will help identify germination factors and determine where a species should emerge and be established^44^. Recent HaloTT model results indicate that osmotic potential, temperature, and their combination, affect GR and GP significantly. A minimum germination was recorded under 0.8 MPa at 20 °C while highest was observed at 30 °C under 0 MPa, suggesting that GP improved with temperature up to 30 °C and reduced with further rise (Fig. 1a–e). Our research findings align with previous studies, indicating that temperature plays a significant role in seed germination. In a large number of plant species, temperature has a significant impact on germination^45^. When the temperature falls below a certain threshold, known as To, a slowed-down rate of water absorption and weakened enzymatic activity are the primary reasons germination timing increases, and germination percentage decreases in seeds^3^. In addition to being sluggish, seed imbibition at low temperatures can also be detrimental to cell membranes, according to Płażek, et al.^46^. Kaur, et al.^47^ observed that cold temperatures reduced chickpea (*Cicer arietinum*) germination, which was linked to increased electrolyte leakage and decreased seed dehydrogenase activity.

GP decreased significantly (p < 0.05) with higher temperatures in the current study. Additionally, the osmotic potential plays an important role in plant germination and establishment^17,48,49^. Furthermore, germination percentage decreases when osmotic potential decreases (− 0.2, − 0.4, 0.6 and − 0.8 MPa). Moreover, the halotime values improved with a rise in cardinal temperature up to optimum temperature, then declined with reduction in the temperature increase beyond the optimum temperature. Additionally, the result of the Ψb of the 50th percentile increased at supraoptimal temperatures^5,42^. Based on the results from a previous study^50^, both GR and GP reduced with a decrease in ψ and rising NaCl at each analyzed T. The GR(g) values significantly increased with reducing Ψ at all cardinal Ts (Table 1).

The cardinal temperatures of seeds, namely the base temperature (Tb), the optimum temperature (To), and the ceiling temperature (Tc), are integral in determining their temperature responses. These cardinal temperatures significantly impact the germination and growth of seeds^3^. According to the current study, the base temperature (Tb) for the investigated plant was 15 °C, below which germination rate declined. Furthermore, the To for maize germination is 33 °C, while the Tc above which plant cannot sustain their biochemical and physiological processes is 45 °C. Furthermore, the previous studies have identified the presence of three cardinal temperatures (cardinal Ts) within the temperature range crucial for seed germination^51^. These cardinal temperatures are indispensable factors in the germination process^3^.

Numerous studies have found comparable Tb values across different plant species. For example, witloof chicory (*Cichorium intybus*) was recorded to have a Tb of 5.3 °C^52^, while chicory (*Cichorium intybus* cv. Grasslands Puna) had a Tb of 3.7 °C^53^. Similarly, rapeseed cultivars were reported to have a Tb range of 0 to 5 °C^54^. In this particular study, the control treatment (0 MPa and 0 M) observed the highest GR50 at 30 °C (To), which aligns with previous findings for chicory, where the maximum GR50 was reported to be between 25 and 30 °C^55^. However, Balandary et al.^56^ recorded a slightly lower value of 25.5 °C using an empirical beta model.

We discovered that percentage of germination and other factors were highest in control (0 MPa) at 30 °C, and lowest at 0.8 MPA at 40 °C. GP and GR are also influenced by temperature, which has been reported previously^49,57,58^. Furthermore, salinity may inhibit germination of seed through osmotic and ion-specific mechanisms^59,60^. Many factors affect seed germination, including salinity, temperature, and water potential. Seeds may germinate at lower osmotic potentials as sodium and calcium ions enter seed cell, decreasing their Ψ and improving embryonic turgor^20^.

According to Liu et al.^43^, the HTT and HaloTT models serve a dual purpose of characterizing the average performance in a seed population and the difference in germination timing among individuals. To forecast germination and timing for a specific environment, it is necessary to record germination times under various environmental conditions. Donohue et al.^61^ further explain that this can be accomplished by calculating the sensitivity threshold value and the total time essential for germination in a seed population. By doing so, we gain a more detailed understanding of the factors contributing to successful germination and can make informed decisions to optimize seed germination in various environments.

Antioxidant enzymes like superoxide dismutase (SOD), peroxidase (POD) and catalase (CAT) level reduces due to water stress. In order to prevent cellular damage, the antioxidant system reduces ROS accumulation by scavenging ROS with enzymes and by increasing antioxidant levels like APX and GPX^62^. SOD is important in the antioxidant defence system because it serves as the first line of defence in scavenging superoxide radicals. During the dismutation of ROS catalyzed by SOD, H_2_O_2_ is produced as a reaction product, which is then scavenged by the activities of CAT and APX^63^. Under water deficiency stress, the levels of ascorbate peroxidase (APX) and guaiacol peroxides (GPX) were reduced. An important antioxidant, ascorbate peroxidase (APX), is responsible for scavenging ROS during oxidative stress. By catalyzing H_2_O_2_ through the reaction of ascorbate peroxidase and utilizing ascorbate as a donor of electrons, ascorbate peroxidase converts H_2_O_2_ into normal water. It is important to note that the expression of APX is regulated differently in response to environmental stresses as well as during normal plant growth and development^64^.

## Conclusions

Using 30 °C and 0 MPa as optimum temperature and osmotic potential, the present study indicates that, the rate of germination and germination percentage were greatly influenced by temperature, osmotic potential and their correlation. The highest Halotime value of 35 degrees Celsius and a maximum R^2^ value of 25 degrees Celsius were observed. Furthermore, σΨb is 0.21 MPa and Ψb (50) is − 0.23 MPa at kT 0.104 MPa. The cardinal temperatures for maize are base temperature = 20 degrees Celsius, optimum temperature = 33 degrees Celsius, and ceiling temperature = 45 degrees Celsius. Therefore, the Halothermal time model correctly described germination time pattern of the studied maize variety.

## Data Availability

All data generated or analyzed during this study are included in this published article.

## Abbreviations

Cl^–^: Chloride ion
GP: Germination percentage
GR: Germination rate, or 1/tg
SG: Seed germination
M: Molar
NaClb(50): The baseline sodium chloride concentration at the 50th percentile
Na +: Sodium ion
HTT: Hydrothermal time model
MPa: Megapascal
NaClb: Median baseline sodium chloride concentration
T: Temperature
HaloTT: Halothermal time model
kT: Temperature slope coefficient for ψb(50) and/or NaClb(50) relative to T above To
NaCl: Sodium chloride concentration (M)"
To: Optimum temperature
Td: The temperature at which ψb(50) and/or NaClb(50) trend begins to change
Tc: Maximum temperature (ceiling temperature)
Tb: Minimum temperature (base temperature)
θH: Hydrotime constant
θHalo: Halotime constant
tg: The time required for a fraction or percentage (g) of seeds to germinate
σNaClb: Standard deviation of NaClb within the seed population
σψb: Standard deviation of ψb within the seed population
TTsub: Thermal time model at sub optimal tempreature
TTsupra: Thermal time model at supra optimal tempreature
